# Evolution of Quality of Life, Functional Capacity, Nutritional Status, and Return to Work in Patients Admitted With Severe COVID-19 Pneumonia Requiring Invasive Mechanical Ventilation: A One-Year Follow-Up Study Post-intensive Care Discharge

**DOI:** 10.7759/cureus.77706

**Published:** 2025-01-20

**Authors:** Melina Carrera, Alejandra Gonzalez, Betiana L Peralta, Ladislao P Diaz Ballve

**Affiliations:** 1 Department of Clinical Medicine/Kinesiology Section, National Hospital Professor Alejandro Posadas, El Palomar, ARG; 2 Department of Clinical Medicine/Pulmonology Service, National Hospital Professor Alejandro Posadas, El Palomar, ARG; 3 Department of Dietetics/Adult Internment Section, National Hospital Professor Alejandro Posadas, El Palomar, ARG; 4 Department of Health, National University of La Matanza, San Justo, ARG

**Keywords:** covid-19, functional status, health related quality of life, nutritional status, postintensive care syndrome, return to work

## Abstract

Background

The COVID-19 pandemic has caused unprecedented challenges in healthcare, particularly for patients with severe cases requiring invasive mechanical ventilation (IMV). Such patients often develop post-intensive care syndrome (PICS), characterized by physical, cognitive, and emotional impairments, severely affecting their health-related quality of life (HRQoL), functional capacity, and reintegration into society.

Objective

This study aimed to evaluate the evolution of HRQoL, functional capacity, nutritional status, and return to work one year after intensive care unit (ICU) discharge in adults with severe COVID-19 who required IMV for over 72 hours.

Methods

A prospective, longitudinal cohort study was conducted between June and December 2020, including 51 patients discharged from the ICU and subsequently followed up for one year. HRQoL was assessed using the EuroQol five-dimension, three-level (EQ-5D-3L) questionnaire and visual analog scale (EQ-VAS). Functional capacity was measured via the Katz Index (KI) and Timed Up and Go (TUG) test. Nutritional status was evaluated through anthropometric measures, including body mass index (BMI) and muscle circumference. Employment reintegration was tracked among pre-hospitalisation workers.

Results

A total of 51 subjects were enrolled in the follow-up program. Only 26 patients (50.9%) attended all scheduled visits. The mean age was 53.4 years, and 34 (68%) were men. A significant improvement was observed in EQ-5D-3L index scores, functional capacity, and muscle strength. Anthropometric indicators increased significantly between one month and one year after hospital discharge. Among those who were previously employed, 11 patients (69.6%) returned to work by the end of the follow-up period, and the overall 1-year mortality was 6 patients (12.2%).

Conclusion

Patients with severe COVID-19 requiring IMV experienced significant impairments in HRQoL, functional independence, and nutritional status at hospital discharge. Over the year-long follow-up, notable improvements were observed in these domains, particularly in muscle strength, anthropometric indicators, and functional outcomes. However, pain and discomfort remained unchanged, reflecting persistent physical sequelae.

With 69.6% of patients returning to work, functional and social recovery was evident, despite lingering limitations. These findings highlight the need for interdisciplinary follow-up programs to support the comprehensive recovery of this population.

## Introduction

The health emergency declared in March 2020 by the World Health Organisation (WHO) led to a high demand for intensive care units (ICUs), with an increase in mortality rates and prolonged hospital stays among patients who survived COVID-19. Most critically ill patients experienced acute respiratory distress syndrome (ARDS) and required invasive mechanical ventilation (IMV). Up to 80% of patients surviving acute respiratory failure after receiving IMV in the ICU develop new or worsening physical, cognitive, and/or mental health impairments, collectively known as post-intensive care syndrome (PICS) [1].

Patients who suffered from COVID-19 and required prolonged ICU stays are at high risk of developing PICS. This is due to the frequent need for IMV, deep sedation, and/or neuromuscular blockade. Moreover, common practices, such as prone positioning and extended ICU stays, further increase the risk of this syndrome [2].

The sequelae following COVID-19 infection, even in mild cases, encompass a wide range of symptoms. Physically, these may include dyspnoea and fatigue. Neurocognitive symptoms, such as anxiety and insomnia, are also frequently observed. These sequelae have been shown to significantly impact health-related quality of life (HRQoL) in affected individuals [3,4].

Another crucial aspect of patients with PICS is the impact on their nutritional status. Prolonged immobility, combined with a hypercatabolic state, has been shown to adversely affect muscle mass in COVID-19 patients requiring mechanical ventilation [5,6].

Weight loss associated with COVID-19 can result from various factors. The NUTRICOVID study observed severe nutritional and functional deterioration among survivors upon hospital discharge [7].

It is important to note that these sequelae and pathological conditions are consequences of critical illness and significantly affect social life, particularly the ability to return to work for those previously employed. In developed countries, 65% of patients typically return to work within a year following critical illness [8]. In our context, among a similar population, only 31% resume employment within the same timeframe [9].

We believe that returning to work or fully resuming previous activities is a milestone in overcoming the impact of critical illness on patients' lives. Full reintegration requires not only physical recovery but also partial or complete psychological and neurocognitive restoration [10].

The aim of this study was to analyze the impact on HRQoL, functional capacity, muscle strength, and nutritional status one year after ICU discharge in adults with COVID-19 who required IMV for more than 72 hours. A secondary objective was to determine the time to return to work and one-year mortality rates.

## Materials and methods

Study design

An analytical, longitudinal, and prospective study was conducted. The Teaching and Research Committee and the Bioethics Committee of the National Hospital Prof. A. Posadas approved the final version of the protocol (Code No. 526LUP0SO/21). This study followed the STROBE guidelines (Strengthening the Reporting of Observational Studies in Epidemiology) to ensure transparency and quality in data presentation. This study is registered with ClinicalTrials.gov (registration number: NCT06727474).

Population and sample

Between June and December 2020, all patients who were discharged alive from the ICU, transferred to the general ward, and subsequently discharged from the hospital were included. Eligible participants were over 18 years old, with an ICU admission diagnosis of COVID-19 pneumonia, confirmed as caused by severe acute respiratory syndrome coronavirus 2 (SARS-CoV-2) through a positive reverse transcription polymerase chain reaction (RT-PCR) test from nasopharyngeal swabs or respiratory tract samples (e.g., tracheal aspirate). Participants had to have received IMV for more than 72 hours, and either the patient or a close relative had to provide informed consent.

Patients were excluded if, at discharge, they had cognitive impairment preventing evaluation of primary outcome variables, had been admitted to the ICU from the penitentiary system, had a history of dementia or cognitive impairment, had a pre-existing tracheostomy, or had pre-ICU indications for prolonged mechanical ventilation at home or in a chronic care facility.

Study variables and data collection

Data were collected using a pre-designed template. To maintain anonymity, patient data were dissociated from their identities, and a unique code was generated for each case, consisting of the initials of the patient's name and surname followed by the last three digits of their national identification number.

Medical records provided information on variables such as age, sex, relevant medical history, APACHE II (Acute Physiology and Chronic Health Evaluation II) score upon ICU admission, significant events during ICU and general ward stays, and employment status before COVID-19 hospitalization, as reported by patients or their families.

The initial patient evaluation, or VISIT 0, occurred after hospital discharge was authorized but before it was effected. This evaluation included HRQoL assessed using the EuroQol five-dimension, three-level questionnaire (EQ-5D-3L), the health status index (EQ-5D-3L Index), and a self-reported visual analog scale (VAS) for perceived health (EQ-VAS), ranging from 0 (worst) to 100 (best).

Muscle strength was assessed using the Medical Research Council Strength Scale (MRC-SS), and functional capacity was measured using the Katz Index (KI).

At one-month post-discharge (VISIT 1), HRQoL, pain, muscle strength, and functional capacity were re-evaluated, with the addition of the Timed Up and Go (TUG) test for independently ambulatory patients. Anthropometric variables, including weight, height, body mass index (BMI), waist circumference (WC), mid-arm circumference (MAC), and calf circumference (CC), were also measured.

At six months post-discharge (VISIT 2), HRQoL, pain, muscle strength, and functional capacity (KI and TUG) were reassessed.

At one year post-discharge (VISIT 3), the same evaluations as VISIT 1 were repeated, including HRQoL, functional capacity, muscle strength, and nutritional variables. In each interview, patients who had been employed before hospitalization were asked about their return to work.

Procedures

The EQ-5D-3L and EQ-VAS questionnaires were administered by the same professional (MC) during each follow-up visit at the outpatient clinic. The EQ-5D-3L includes five descriptive dimensions (mobility, self-care, usual activities, pain/discomfort, and anxiety/depression). Responses were used to derive a health state and calculate the EQ-5D-3L Index. The VAS allowed participants to rate their health from 0 (worst imaginable) to 100 (best imaginable).

Functional capacity was assessed using the KI questionnaire and the TUG test, which recorded the time taken for participants to rise from a chair, walk three meters, and return. The time was measured in seconds.

Muscle strength (MRC-SS) was assessed with patients in a supine position, with the same verbal instructions used for each muscle group assessment.

Anthropometric measurements were performed by the same investigator (LP) during VISIT 1 and VISIT 3. Measurements were taken using a non-extensible tape measure. MAC was measured at the midpoint between the acromion and olecranon with the arm flexed at 90°, then averaged between both sides. CC was measured at the largest circumference of the calf with the leg extended or slightly flexed in patients with limited mobility.

Patients were questioned about their living conditions and return to work. For those who missed a visit, medical records were reviewed, and follow-up phone calls were made to confirm survival.

Patients attended follow-up clinics, which included clinical and pulmonology evaluations. If the study investigators (MC and LP) detected health issues during visits, the clinic's coordinating physician was informed of appropriate follow-up.

Statistical analysis

Descriptive analyses summarised categorical variables as absolute frequencies and percentages. Numerical variables were presented as measures of central tendency (mean or median) and dispersion (standard deviation) or position (25th and 75th percentiles) as appropriate. The Kolmogorov-Smirnov test assessed normality in the complete sample (n=51), and the Shapiro-Wilk test was used for smaller groups.

Comparisons of outcome variables across visits used repeated measures analysis of variance (ANOVA) or Friedman tests as appropriate. For global comparisons with significant differences, Bonferroni or Dunn-Bonferroni tests identified significant pairs. For nutritional status comparisons assessed twice, the student’s t-test or Wilcoxon signed-rank test was used, as appropriate.

A p-value < 0.05 was considered significant for all hypothesis tests. Analyses were conducted using IBM SPSS for Windows, version 25.0 (IBM Corp, Armonk, NY, USA), R software version 3.6.3 (R Core Team, 2020; R Foundation for Statistical Computing, Vienna, Austria), and the ggplot2 package version 3.3.2 (Wickham, 2016) for graphical presentations.

## Results

**Figure 1:**
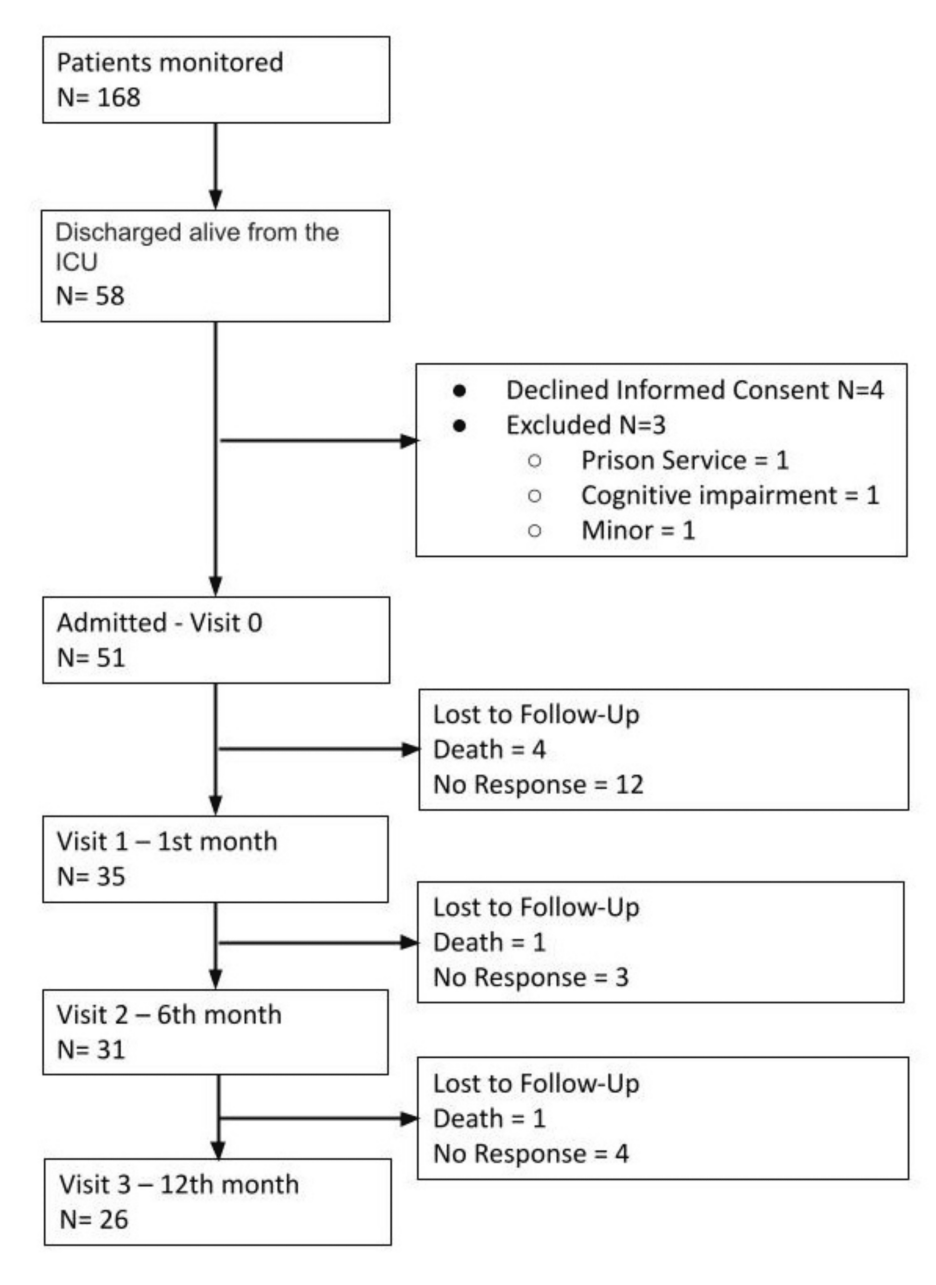
Flowchart of patients under follow-up

Between June and December 2020, 168 patients with COVID-19 were admitted to the ICU and required IMV. Of these, 58 were discharged alive from the ICU, and 51 entered the follow-up program. Only 26 patients (50.9%) attended all scheduled visits, which included assessments at one month, six months, and one year post-hospital discharge (Figure 1).

The clinical and demographic characteristics of the 51 included patients are presented in Table 1.

**Table 1:** Characteristics of the sample upon admission to the inpatient ward X: mean; SD: standard deviation; Md: median; (p25-p75): 25th-75th percentiles; *n=16 APACHE: Acute Physiology and Chronic Health Evaluation

	n=51
Male sex, n (%)	34 (66.6)
Age in years, x (SD)	53.43 (10.94)
Comorbidities	
Hypertension, n (%)	22 (43.1)
Diabetes, n (%)	15 (29.4)
Obesity, n (%)	13 (25.4)
Smoking, n (%)	6 (11.7)
History of respiratory disease, n (%)	3 (5.8)
Weight at discharge in Kg, x (SD)	75.1 (19)
Height in cm, x (DE)	162 (0.08)
Body mass index (BMI) in kg/m^2^, x (SD)	27.4 (7.1)
BMI categories, n (%)	
less than 18.5 kg/m^2^	0 (0)
between 18.5 and 24.9 kg/m^2^	13 (44.8)
between 25 and 29.9 kg/m^2^	10 (34.5)
30 kg/m^2^ or more	6 (20.7)
Charlson Score, Md (p25 - p75)	2 (0.25 - 2)
APACHE score upon ICU admission, Md (p25 - p75)	12 (10 - 17)
Days on invasive mechanical ventilation, Md (p25 - p75)	17 (10 - 35)
Patients who received prone position, n (%)	31 (60.7)
Days from intubation (IOT) to tracheostomy (TQT)T, Md (p25 - p75)	23 (20 - 28)
Patients who required TQT, n (%)	16 (31.3)
Patients decannulated in the ICU, n (%)	12 (75.0)*
ICU length of stay, Md (p25 - p75)	22 (13 - 40)
Hospital length of stay, Md (p25 - p75)	45 (27 - 65)
Patients deceased at 12 months, n (%)	6 (12.2)

**Figure 2:**
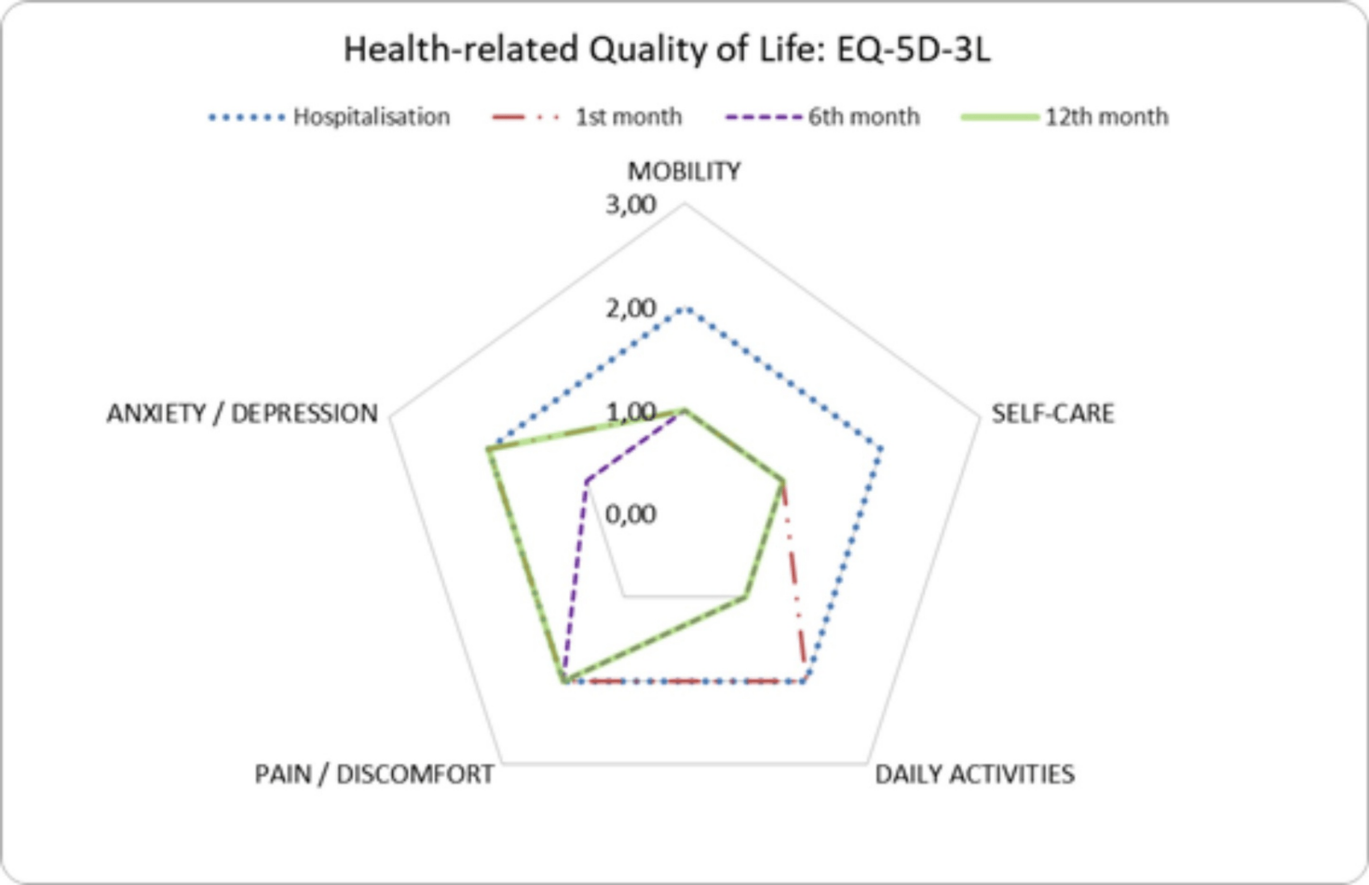
Changes in EQ-5D-3L domains according to the visit EQ-5D-3L: EuroQol five-dimension, three-level questionnaire

Table 2 presents the main outcome variables: HRQoL, functional capacity, and muscle strength. A significant improvement was observed in the EQ-5D-3L index values. The improvement in the index value can be attributed to better mobility, self-care, daily living activities (DLA), and anxiety scores, all of which showed significant differences across evaluations. The pain and discomfort domain remained constant throughout the follow-up period (Figure 2).

**Table 2:** Evolution of health-related quality of life, functional independence, and muscle strength during follow-up HRQOL: Health-related quality of life; EQ-5D-3L: EuroQol-5 dimensions - 3 levels; The subscripts a, b, c, d that differ between columns denote differences between pairs in the post-hoc analysis using the Dunn-Bonferroni test; VAS: visual analog scale; MRC: medical research council †: For six patients, discharge was completed without the possibility of evaluation. *: all values are expressed as medians (25th - 75th percentiles)

	Hospitalization	1st month	6th month	12th month	Absolute difference	
	n = 45 (†)	n = 35	n = 31	n = 26		p*
HRQOL*						
EQ-5D-3L Mobility	2 (1 - 3)	2 (1 - 2)	1 (1 - 1)	1 (1 - 1)	1	<0.001
	a	a.b.c.d	b.c.d	b.c.d		
EQ-5D-3L Personal Care	2 (1 - 3)	1.5 (1 - 2)	1 (1 - 1)	1 (1 - 1)	1	<0.001
	a	a.b.c.d	b.c.d	b.c.d		
EQ-5D-3L Daily Activities	2 (1 - 3)	2 (1 - 2)	1 (1 - 2)	1 (1 - 1)	1	<0.001
	a	a	a.b	b		
EQ-5D-3L Pain and Discomfort	2 (1 - 3)	2 (1 - 2)	2 (1 - 2)	2 (1 - 2)	0	0.216
	a	a	a	a		
EQ-5D-3L Anxiety and Depression	2 (1 - 3)	2 (1 - 2)	1 (1 - 2)	1.5 (1 - 2)	1	0.01
	a	a.b.c	b.d	c.d		
EQ-5D-3L Index	0.45 (0.22- 0.57)	0.56 (0.49 - 0.72)	0.74 (0.55 - 1)	0.73 (0.59 - 1)	0.29	<0.001
	a	a.b.c	b.c.d	d		
Self-Perception of Health EQ-5D-3L-VAS	70 (50 - 82.5)	80 (70 - 82.5)	80 (70 - 86.2)	80 (70 - 90)	10	0.183
	a	a	a	a		
Functional Independence*						
Katz index	6 (4.75 - 6)	1.5 (1 - 2.5)	1 (1 -1)	1 (1 -1)	5	<0.001
	a	b	b	b		
Timed Up to Go Test	NO	10.9 (8.7 - 17.9)	9.1 (7.2 - 12.5)	8.5 (7.3 - 9.3)	2.4	0.006
		a	a.b	b		
Muscle Strength*						
MRC Strength Scale	52 (40.5 - 56.2)	59 (54 - 60)	60 (60 - 60)	60 (57 - 60)	8	<0.002
	a	b	b	b		
Weakness Acquired in the ICU, n/n Total (%)	15/46 (32.6)	6/38 (15 - 8)	1/22 (4 - 5)	0/0 (0)		

Functional capacity, measured by the KI, showed significant improvement between the initial and subsequent evaluations. Similarly, improvements were observed in the p-TUG scores, with significant changes between the initial assessment at one month and the final measurement at 12 months.

Lastly, significant changes were observed in muscle strength measured with the EF-MRC scale. Starting with a median score of 52 points (40.7-56.2) at hospital discharge, significant changes were observed in the global comparison. A marked improvement was noted between discharge and the second evaluation. Subsequent assessments showed slight, non-significant improvements, reaching the ceiling score of 60 points by the third visit (sixth month) onward.

Table 3 shows changes in anthropometric indicators, with all measured variables significantly increasing between one month and one year post-hospital discharge.

**Table 3:** Evolution of anthropometric variables between the month and one year after hospital discharge *Mean (SD); †student's T-test for paired samples

	1st month	12th month	Mean difference	p^†^
Weight difference in %	-5.2 (3.3)*	-15.2 (11.6)*	10	0.001
Body mass index (kg/m2)	31.0 (7.7)*	34.6 (7.9)*	-3.6	<0.001
Waist circumference (cm)	105.6 (11.9)*	114.9 (15.8)*	-9.4	<0.001
Arm perimeter (cm)	30.8 (5.1)*	34.4 (4.7)*	-3.6	<0.001
Calf perimeter (cm)	34.5 (5.1)*	39.0 (4.8)*	-4.5	<0.001

Table 4 displays the follow-up results for 25 patients who completed one year of observation. Among this group, 7 (38.8%) patients of those employed prior to ICU admission had not returned to work by the end of the follow-up period.

**Table 4:** Description of patients with prior employment before admission and their reintegration

Previously worked	N=18
Did not return after a year	7 (38.8%)
Returned before the year	11 (69.6%)
Returned before the 1st month	2 (18.1%)
Returned before the 6th month	6 (54.5%)
Returned before the 12th month	3 (27.2%)

Among the 51 discharged patients, 6 (11.7%) died during the first year of follow-up. A detailed analysis of these deaths reveals that 4 (7.8%) patients died within the first month after discharge, 1 (1.9%) died between the first and second month of follow-up (visit 2), and the last 1 (1.9%) died before completing the year of follow-up.

## Discussion

We observed that the HRQoL reported by patients at hospital discharge showed a significant overall deterioration (EQ-5D-3L index). However, a marked improvement was noted during follow-up. It is worth highlighting that these results differ from those reported for the general population by Janssen [11]. In similar studies, such as that by Argento et al., a higher index was observed in post-COVID-19 patients who had been in the ICU. However, their sample was more heterogeneous, as it included both patients who received IMV and those who did not [12].

Self-perceived quality of life (EQ-5D-3L - VAS) also showed improvement from hospital discharge to the one-year follow-up. This variable exhibited a similar trend in recent studies involving comparable populations [13,14].

When compared to normal values for the Argentinian population, the one-year follow-up in our series reached a median value equal to those reported in the literature. Notably, our series began with self-reported quality of life ratings between "fair" and "poor" and ended with values between "good" and "very good" [15].

Examining each EQ-5D-3L domain individually, we observed that by one year, most patients had no difficulties walking, performing self-care tasks, or carrying out daily activities. The only domain that showed no significant changes throughout the year was pain and discomfort. This finding aligns with other studies that reported persistent issues in this domain after one year of follow-up [12-14].

In our study, functional independence was assessed using the Katz Index (KI) and the TUG test. Regarding the KI, an improvement was noted over time, although full independence was not achieved in most cases. This trend is similar to that reported by Schmidt et al., who followed COVID-19 patients for six months post-ICU discharge, and their series also failed to achieve complete functional independence [16]. Similarly, Latronico et al. reported that 98% of patients regained independence in ADLs at three months, with 100% achieving it at one year. However, these authors opted to measure functional independence using the Barthel Index, which has a higher ceiling effect, limiting its utility in patients with high initial functionality levels [17].

In patients with greater independence, physical tests, such as the TUG test, are more precise in detecting improvements. In our series, TUG test results improved significantly across evaluations, consistent with findings by Shizuo et al., who followed post-COVID-19 patients for one year and observed an improvement of up to 6.6 ± 1.7 seconds. However, unlike our study, those authors included patients who underwent a weekly rehabilitation program of three hours over six weeks, which could have influenced recovery [18].

Intensive care unit-acquired weakness (ICUAW) is a high-incidence event in this population [19]. In our sample, muscle strength was evaluated using the MRC scale, showing an initial deterioration but a progressive recovery. The most significant improvement was observed between the pre-discharge evaluation and the one-month follow-up. By 12 months, none of the evaluated patients exhibited muscle weakness. These results align with findings by Latronico et al., although their follow-up began three months after discharge [17].

Regarding anthropometric variables, our observations are consistent with those of other studies. Although initial measurements were taken at one month, all patients exhibited some degree of malnutrition. All variables improved by one year, possibly due to a synergy between multidisciplinary follow-up and the fact that patients started with lower-than-usual parameters [14,20,21].

We believe the ultimate milestone of functional independence is re-employment. In patients admitted to the ICU for severe COVID-19, the return-to-work rate varies considerably, ranging from 61% to 88% in different studies. In our investigation, 69.6% of patients were able to return to their previous employment [2,13,17,18].

In our cohort of 51 discharged patients, we observed a one-year mortality rate of 11.7%. This is higher than the 7.3% reported in a similar study with an 18-month follow-up [12]. In contrast, Zangrillo et al. did not record any deaths at the one-year follow-up [14]. Differences in outcomes may be partly attributed to our study’s inclusion criteria, which consisted exclusively of patients requiring IMV for at least 72 hours.

Finally, this study is not without limitations. A key issue is the high dropout rate, which may be explained by socioeconomic factors and mobility problems that prevented some patients from attending scheduled follow-ups. Additionally, the patients included in this study belonged to the first wave of the pandemic. The onset of the second wave complicated patient follow-ups, with several reporting fear of re-infection as a reason for absence. On the other hand, an encouraging but limiting factor for data collection was that many patients, having achieved recovery and greater independence, may have felt it unnecessary to return for follow-up consultations after discharge.

## Conclusions

Patients with severe COVID-19 requiring invasive mechanical ventilation experienced significant impairments in health-related quality of life, functional independence, and nutritional status at hospital discharge. Over the year-long follow-up, notable improvements were observed in these domains, particularly in muscle strength, anthropometric indicators, and functional outcomes. However, pain and discomfort remained unchanged, reflecting persistent physical sequelae.

With 69.6% of patients returning to work, functional and social recovery was evident, despite lingering limitations. These findings highlight the need for interdisciplinary follow-up programs to support the comprehensive recovery of this population.

## References

[1] Needham DM, Davidson J, Cohen H (2012). Improving long-term outcomes after discharge from intensive care unit. Report from a stakeholders' conference. Crit Care Med.

[2] Demoule A, Morawiec E, Decavele M (2022). Health-related quality of life of COVID-19 two and 12 months after intensive care unit admission. Ann Intensive Care.

[3] Carfì A, Bernabei R, Landi F (2020). Persistent symptoms in patients after acute COVID-19. JAMA.

[4] Wang X, Xu H, Jiang H (2020). Clinical features and outcomes of discharged coronavirus disease 2019 patients: a prospective cohort study. QJM.

[5] Pironi L, Sasdelli AS, Ravaioli F (2021). Malnutrition and nutritional therapy in patients with SARS-CoV-2 disease. Clin Nutr.

[6] Whittle J, Molinger J, MacLeod D, Haines K, Wischmeyer PE (2020). Persistent hypermetabolism and longitudinal energy expenditure in critically ill patients with COVID-19. Crit Care.

[7] Álvarez-Hernández J, Matía-Martín P, Cáncer-Minchot E, Cuerda C (2023). Long-term outcomes in critically ill patients who survived COVID-19: the NUTRICOVID observational cohort study. Clin Nutr.

[8] Kamdar BB, Suri R, Suchyta MR (2020). Return to work after critical illness: a systematic review and meta-analysis. Thorax.

[9] Das Neves AV, Vasquez DN, Loudet CI (2015). Symptom burden and health-related quality of life among intensive care unit survivors in Argentina: a prospective cohort study. J Crit Care.

[10] Schwitzer E, Jensen KS, Brinkman L (2023). Survival ≠ recovery. A narrative review of post-intensive care syndrome. CHEST Critical Care.

[11] Janssen MF, Szende A, Cabases J, Ramos-Goñi JM, Vilagut G, König HH (2019). Population norms for the EQ-5D-3L: a cross-country analysis of population surveys for 20 countries. Eur J Health Econ.

[12] Argento F, Donato M, Villalba D, Sarubbio MG, Giménez A, Ciapponi A, Augustovski F (2024). Mortalidad, Secuelas Clínicas y Calidad de Vida Luego del Alta de Unidades de Cuidados Intensivos en Pacientes con COVID-19: Estudio Multicéntrico Descriptivo en Argentina [Article in Spanish]. Value Health Reg Issues.

[13] Huang L, Yao Q, Gu X (2021). 1-year outcomes in hospital survivors with COVID-19: a longitudinal cohort study. Lancet.

[14] Zangrillo A, Belletti A, Palumbo D (2022). One-year multidisciplinary follow-up of patients with COVID-19 requiring invasive mechanical ventilation. J Cardiothorac Vasc Anesth.

[15] Augustovski F, Rey-Ares L, Gibbons L (2013). Atlas Argentino de Calidad de Vida Relacionada con la Salud: Análisis de los datos de la Encuesta Nacional de Factores de Riesgo por Provincias [Article in Spanish]. Value Health Reg Issues.

[16] Schmidt D, Margarites AG, Alvarenga LP, Paesi PM, Friedman G, Sbruzzi G (2023). Post-COVID-19 intensive care unit-acquired weakness compromises long-term functional status. Phys Ther.

[17] Latronico N, Peli E, Calza S (2022). Physical, cognitive and mental health outcomes in 1-year survivors of COVID-19-associated ARDS. Thorax.

[18] Ida FS, Ferreira HP, Vasconcelos AK, Furtado IA, Fontenele CJ, Pereira AC (2024). Post-COVID-19 syndrome: persistent symptoms, functional impact, quality of life, return to work, and indirect costs - a prospective case study 12 months after COVID-19 infection. Cad Saude Publica.

[19] Diaz Ballve LP, Dargains N, Urrutia Inchaustegui JG (2017). Weakness acquired in the intensive care unit. Incidence, risk factors and their association with inspiratory weakness. Observational cohort study. Rev Bras Ter Intensiva.

[20] Leggieri C, Dezza L, Oltolini B (2021). Long-term quality of life after intensive care unit admission (a single-center observational study). Gen Reanimatol.

[21] Rovere Querini P, De Lorenzo R, Conte C (2020). Post-COVID-19 follow-up clinic: depicting chronicity of a new disease. Acta Biomed.

